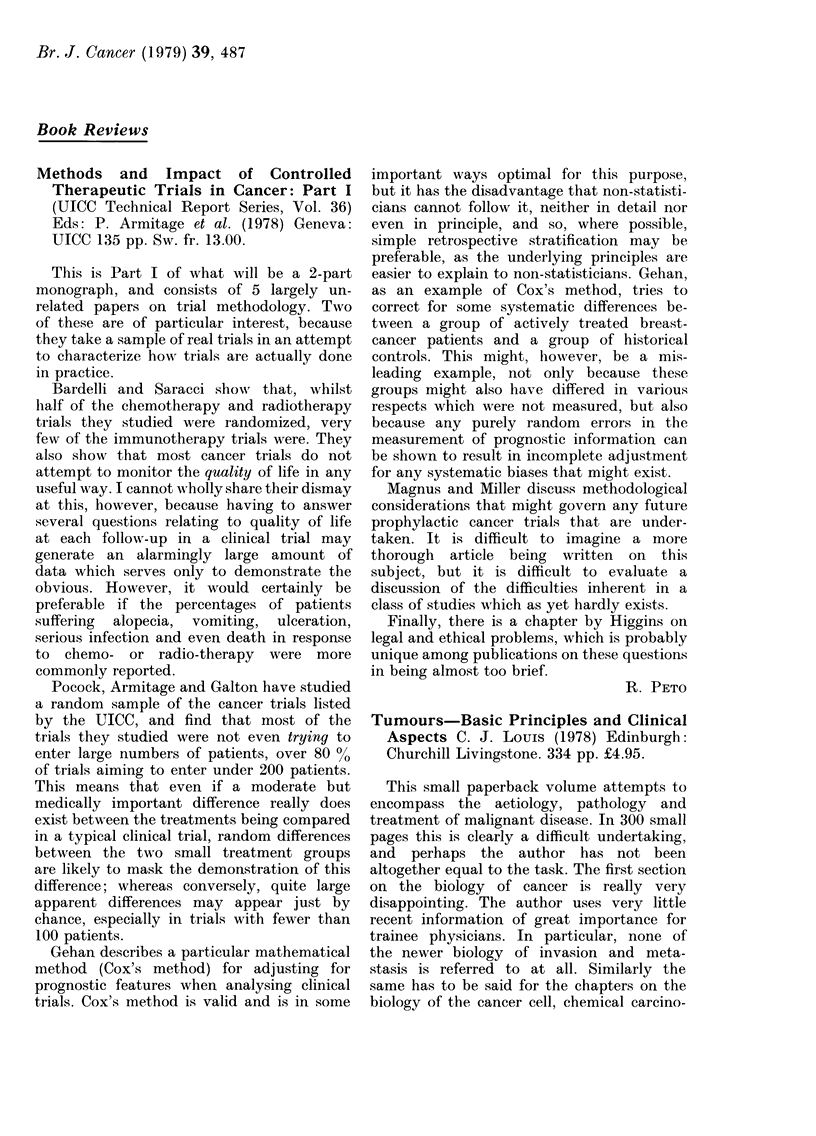# Methods and Impact of Controlled Therapeutic Trials in Cancer: Part I

**Published:** 1979-04

**Authors:** R. Peto


*Br. J. Cancer* (1979) **39**, 487

### *Book Reviews*

**Methods and Impact of Controlled Therapeutic Trials in Cancer: Part I** (UICC Technical Report Series, Vol. 36) Eds: P. Armitage *et al.* (1978) Geneva: UICC 135 pp. Sw. fr. 13.00.

This is Part I of what will be a 2-part monograph, and consists of 5 largely unrelated papers on trial methodology. Two of these are of particular interest, because they take a sample of real trials in an attempt to characterize how trials are actually done in practice.

Bardelli and Saracci show that, whilst half of the chemotherapy and radiotherapy trials they studied were randomized, very few of the immunotherapy trials were. They also show that most cancer trials do not attempt to monitor the *quality* of life in any useful way. I cannot wholly share their dismay at this, however, because having to answer several questions relating to quality of life at each follow-up in a clinical trial may generate an alarmingly large amount of data which serves only to demonstrate the obvious. However, it would certainly be preferable if the percentages of patients suffering alopecia, vomiting, ulceration, serious infection and even death in response to chemo- or radio-therapy were more commonly reported.

Pocock, Armitage and Galton have studied a random sample of the cancer trials listed by the UICC, and find that most of the trials they studied were not even *trying* to enter large numbers of patients, over 80 % of trials aiming to enter under 200 patients. This means that even if a moderate but medically important difference really does exist between the treatments being compared in a typical clinical trial, random differences between the two small treatment groups are likely to mask the demonstration of this difference; whereas conversely, quite large apparent differences may appear just by chance, especially in trials with fewer than 100 patients.

Gehan describes a particular mathematical method (Cox's method) for adjusting for prognostic features when analysing clinical trials. Cox's method is valid and is in some important ways optimal for this purpose, but it has the disadvantage that non-statisticians cannot follow it, neither in detail nor even in principle, and so, where possible, simple retrospective stratification may be preferable, as the underlying principles are easier to explain to non-statisticians. Gehan, as an example of Cox's method, tries to correct for some systematic differences between a group of actively treated breast-cancer patients and a group of historical controls. This might, however, be a misleading example, not only because these groups might also have differed in various respects which were not measured, but also because any purely random errors in the measurement of prognostic information can be shown to result in incomplete adjustment for any systematic biases that might exist.

Magnus and Miller discuss methodological considerations that might govern any future prophylactic cancer trials that are undertaken. It is difficult to imagine a more thorough article being written on this subject, but it is difficult to evaluate a discussion of the difficulties inherent in a class of studies which as yet hardly exists.

Finally, there is a chapter by Higgins on legal and ethical problems, which is probably unique among publications on these questions in being almost too brief.

R. Peto